# Bioinformatics analysis and experimental validation of a novel autophagy-related signature relevant to immune infiltration for recurrence prediction after curative hepatectomy

**DOI:** 10.18632/aging.204632

**Published:** 2023-04-03

**Authors:** Huaxiang Wang, Chengkai Yang, Dong Li, Ruling Wang, Yanbing Li, Lizhi Lv

**Affiliations:** 1 Department of Hepatobiliary Surgery, Fuzong Clinical Medical College of Fujian Medical University, Fuzhou, Fujian 350025, China; 2 Department of Hepatobiliary Surgery, 900 Hospital of the Joint Logistic Team, Fuzhou, Fujian 350025, China; 3 Department of Hepatobiliary and pancreatic Surgery, Taihe Hospital, Affiliated Hospital of Hubei University of Medicine, Shiyan, Hubei 442000, China; 4 Department of Anesthesiology, Hubei Provincial Hospital of Traditional Chinese Medicine, Wuhan, Hubei 430061, China

**Keywords:** gene signature, hepatocellular carcinoma, genetic alteration, SNRPE, immune infiltrates, prognostic value

## Abstract

Hepatocellular carcinoma (HCC) remains imposing an enormous economic and healthcare burden worldwide. In this present study, we constructed and validated a novel autophagy-related gene signature to predict the recurrence of HCC patients. A total of 29 autophagy-related differentially expressed genes were identified. A five-gene signature (CLN3, HGF, TRIM22, SNRPD1, and SNRPE) was constructed for HCC recurrence prediction. Patients in high-risk groups exhibited a significantly poor prognosis compared with low-risk patients both in the training set (GSE14520 dataset) and the validation set (TCGA and GSE76427 dataset). Multivariate cox regression analysis demonstrated that the 5-gene signature was an independent risk factor for recurrence-free survival (RFS) in HCC patients. The nomograms incorporating 5-gene signature and clinical prognostic risk factors were able to effectively predict RFS. KEGG and GSEA analysis revealed that the high-risk group was enriched with multiple oncology characteristics and invasive-related pathways. Besides, the high-risk group had a higher level of immune cells and higher levels of immune checkpoint-related gene expression in the tumor microenvironment, suggesting that they might be more likely to benefit from immunotherapy. Finally, the immunohistochemistry and cell experiments confirmed the role of SNRPE, the most significant gene in the gene signature. SNRPE was significantly overexpressed in HCC. After SNRPE knockdown, the proliferation, migration and invasion ability of the HepG2 cell line were significantly inhibited. Our study established a novel five-gene signature and nomogram to predict RFS of HCC, which may help in clinical decision-making for individual treatment.

## INTRODUCTION

In recent years, molecular targeted therapy and immunotherapy have been extensively used in cancer treatment. However, the prognosis of hepatocellular carcinoma (HCC) patients has not been significantly improved [1, 2]. Recent data suggested that 5 years overall survival (OS) rate of all stages of HCC patients was only 20%, while 17% in 2016 [3, 4]. The poor prognosis of HCC patients was owing to the high recurrence. Previous studies suggested that more than 70% recurrence occurred within five years in patients who have undergone radical resection [5–7]. Hence, the identification of an effective biomarker for recurrence prediction of HCC patients was essential for prognosis improvement. IL-11, STAT3, AFP, C14orf166, HNRNPA2B1, GP73, and CTSA have been suggested as candidate recurrence prediction biomarkers in previous studies [8–12]. But even the gold standard biomarker, alpha-fetoprotein, was only elevated in 70% of HCC patients [13]. The sensitivity of multi-gene recurrence predicting markers that was higher than a single biomarker has been broadly accepted, especially combined with the clinical characteristics.

Autophagy, another process of programmed death that is different from apoptosis, plays a dual role in many types of cancer [14]. In the stage of tumorigenesis, autophagy inhibited tumor progression by eliminating damaged proteins and organelles. However, once the tumor formed, autophagy promoted tumor cell proliferation and metastasis by stimulating cell metabolism [15, 16]. The abnormal expression of autophagy-related genes (ARGs) plays a key role in regulating the process of autophagy. Wei Q et al. reported that E2-induced activation of the NLRP3 inflammasome inhibited HCC progression by promoting autophagy [17]. Quidville and colleagues reported that after siRNA depleted the components of the core spliceosome (such as SNRPE or SNRPD1), autophagy was triggered in breast and lung cancer cell lines, and cell viability was significantly reduced [18]. In addition, inhibition of the autophagy regulated by autophagy gene autophagy-related 7 (ATG7) can cause the accumulation of damaged mitochondria and reactive oxygen species (ROS), resulting in inhibition of cancer recurrence [19]. Hence, ARGs have become the potential effective biomarker for therapy and recurrence prediction. However, there is still a lack of studies on the establishment of multi-ARGs signatures to predict recurrence.

We constructed a five ARGs signature using the RNA expression data and clinical data from the GSE14520 dataset in the GEO database. We validated the availability of genes signature using the independent HCC cohort in the TCGA and GSE76427 dataset. Then, we constructed a nomogram integrating genes signature and all independent risk factors of (recurrence-free survival) RFS to further explore the recurrence predicting model to acquire the clinical net benefit that effectively guides the clinical decision. In addition, we performed the function analysis by GO, KEGG, and GSEA to explore the potential mechanism of these ARGs. We assessed the affection of five genes signature on immune infiltration in the CIBERSORT algorithm and TIMER database. Finally, the immunohistochemistry and cell experiments confirmed the role of SNRPE, the most significant gene in the gene signature.

## RESULTS

### Identification of differential expressed ARGs in HCC

**Figure 1:**
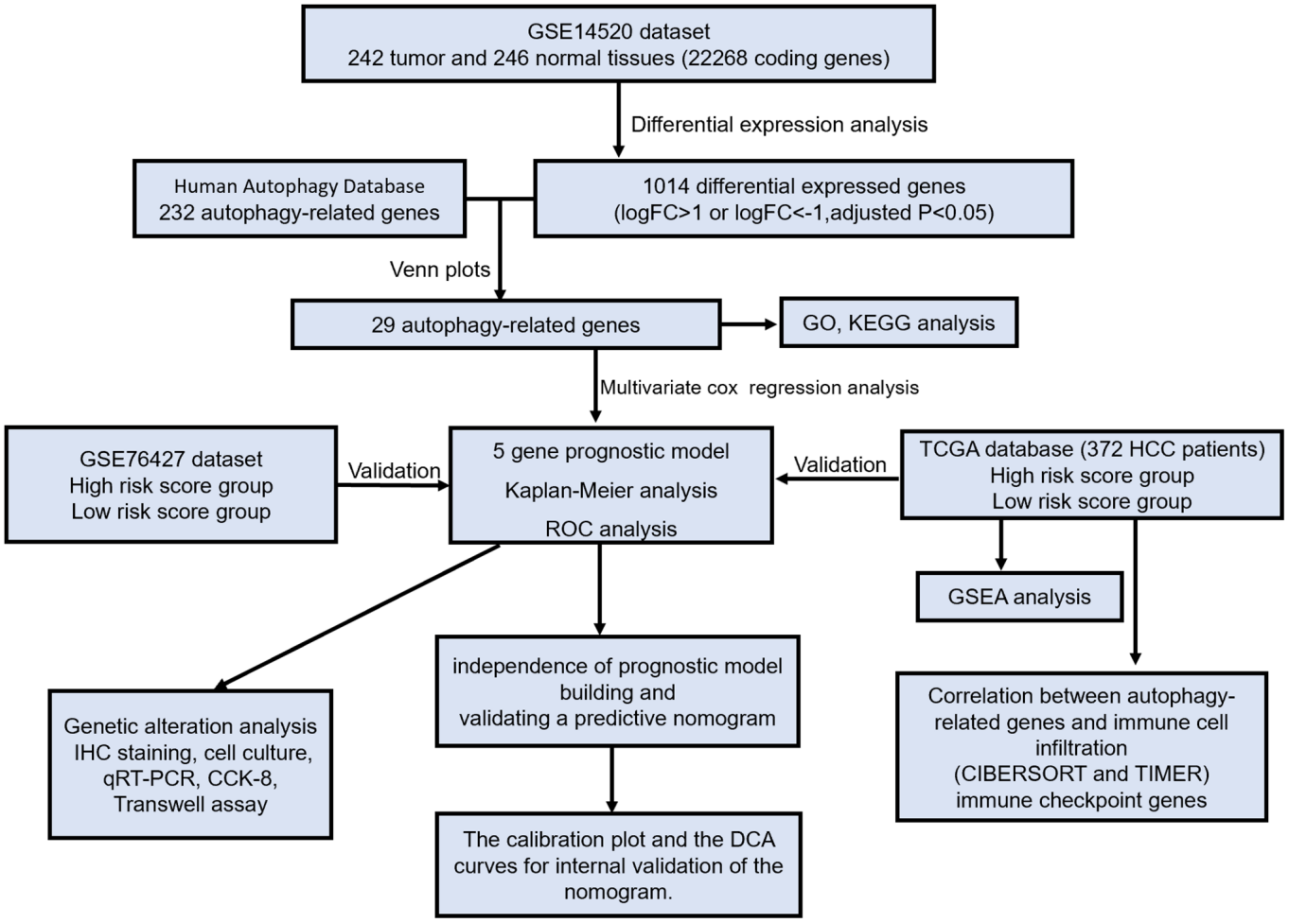
The flow chart exhibited the concise scheme of our study on autophagy-related gene signatures and combined nomogram model for predicting recurrence-free survival of hepatocellular carcinoma patients.

A flow chart was established to exhibit a concise scheme of our study (Figure 1). We identified 1014 differential expressed genes (575 up-regulated and 539 down-regulated genes) in 242 HCC tissues compared with 246 non-HCC tissues from the GSE14520 dataset. Then, 232 ARGs were identified from the HADb and PubMed. Next, we screened out 29 overlapping genes from 1014 differential expressed genes and 232 ARGs for subsequent analysis.

### Generation of the prognostic five-gene signature

We performed the Univariate and Multivariate Cox proportional hazards regression analysis sequentially on 29 differentially expressed ARGs to obtain genes that independently predict the RFS of HCC patients. Finally, five genes were identified to construct a predictive gene signature. The five genes were ceroid-lipofuscinosis, neuronal 3 (CLN3), hepatocyte growth factor (HGF), tripartite motif-containing 22 (TRIM22), small nuclear ribonucleoprotein Sm D1 (SNRPD1), and small nuclear ribonucleoprotein polypeptide E (SNRPE). The risk score was accordingly calculated following: Risk score = (−0.346) × expression_CLN3_ + 0.459 × expression_HGF_ + (−0.233) × expression_TRIM22_ + 0.258 × expression_SNRPD1_ + 0.139 × expression_SNRPE_ (Table 1).

**Table 1:** Multivariate Cox regression analysis of the 5-gene signature in the GSE14520 dataset.

**Gene**	**Coef**	**aHR**	**Lower 95% CI**	**Upper 95% CI**	^*^P-Value
CLN3	−0.346	0.708	0.503	0.996	0.047
HGF	0.459	1.582	1.244	2.013	<0.001
TRIM22	−0.233	0.792	0.668	0.939	0.007
SNRPD1	0.258	1.294	1.023	1.639	0.032
SNRPE	0.139	1.719	1.102	1.986	0.029

Abbreviations: aHR: adjusted hazard ratio; CI: confidence interval.

### Validation of the prognostic five-gene signature in training set

**Figure 2:**
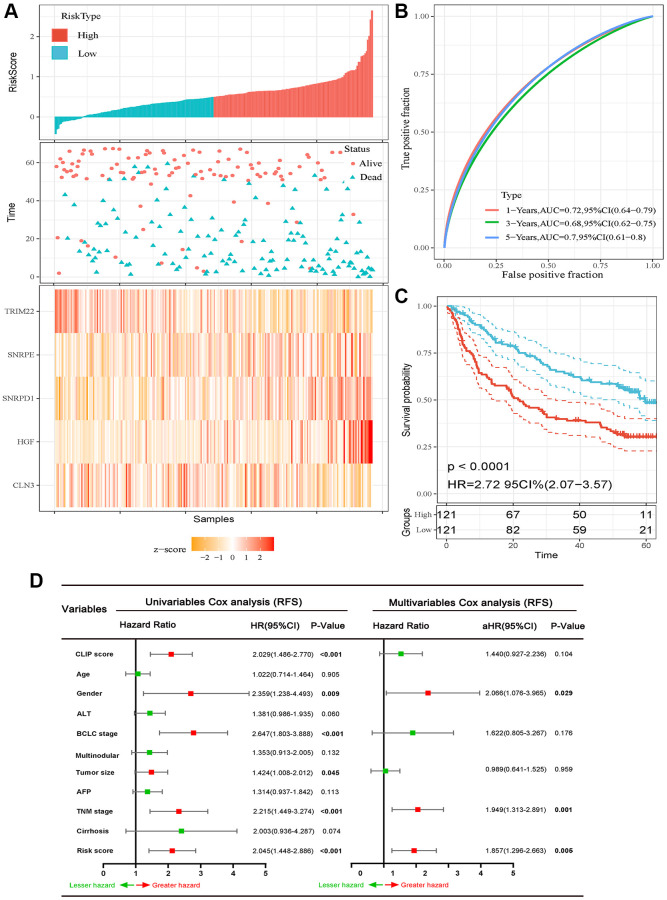
**Prognostic analysis of the five-autophagy-related gene signature model in the training set (GSE14520 dataset).** (**A**) Risk score and mRNA expressed heatmap of the five-gene signature. (**B**) Time-dependent ROC curves of the five-gene signature in the training set. (**C**) High-risk score correlated with poor RFS probability in the training set. (**D**) Results of the univariate and multivariate Cox regression analyses regarding of RFS in the training set.

We calculated the risk score for each HCC patient in the GSE14520 dataset and then classified the 242 HCC patients into high-risk score group and low-risk score group taking the cutoff value of the median risk score (Figure 2A). AUCs of ROC curves for 1-, 3-, and 5-year RFS were 0.72, 0.68, and 0.70, respectively (Figure 2B). In addition, the K-M curve revealed that patients in high-risk score subgroups have shorter RFS (Figure 2C). We also illuminated the association between risk score and clinicopathological parameters and demonstrated that high-risk score correlated to increased TNM stage (*P* = 0.020), serum AFP level (*P* = 0.049), multinodular (*P* = 0.012), CLIP score (*P* = 0.037), BCLC stage (*P* = 0.005), recurrence rate (*P* < 0.001), and lower alive rate (*P* = 0.002) (Table 2). Furthermore, as shown in Figure 2D, the multivariate Cox regression analyses revealed that gender (aHR (95% CI): 2.006 (1.076−3.965); *P* = 0.029), TNM stage (aHR (95% CI): 1.949 (1.313–2.891); *P* = 0.001), and high-risk score (aHR (95% CI): 1.857 (1.296–2.663); *P* = 0.005) were independent predictors for RFS (Figure 2D).

**Table 2:** Correlation between risk score and clinicopathological features of HCC patients for RFS in the GSE14520 dataset.

**Characteristics**		***N***	**Risk score level**		***X* ^2^**	^*^P-Value
			**Low**	**High**		
Age	≥55	76	34	42	1.329	0.249
	<55	166	87	79		
Gender	Male	211	105	106	0.037	0.847
	Female	31	16	15		
Main tumor size	≥5 cm	88	40	48	1.143	0.285
	<5 cm	154	81	73		
TNM stage	I–II	174	97	77	5.400	0.020
	III–IV	51	19	32		
Serum AFP level	≥300 ng/ml	110	47	63	3.842	0.049
	<300 ng/ml	128	71	57		
ALT	≥50 U/L	100	44	56	2.454	0.117
	<50 U/L	142	77	65		
Multinodular	Yes	52	18	34	6.270	0.012
	No	190	103	87		
Cirrhosis	Yes	223	109	114	1.428	0.232
	No	19	12	7		
CLIP score	≥2	52	20	32	4.331	0.037
	<2	173	95	78		
BCLC stage	B–C	53	18	35	7.934	0.005
	0–A	173	97	76		
Recurrence	Yes	136	54	82	13.161	<0.001
	No	106	67	39		
Survival status	Dead	96	36	60	9.945	0.002
	Alive	146	85	61		

Abbreviations: TNM: tumor, node, metastasis; AFP: alpha fetoprotein; ALT: alanine aminotransferase; CLIP: Cancer of the Liver Italian Program score; BCLC: Cancer of the Liver Italian Program score. ^*^P-Value < 0.05 were considered statistically significant.

### Validation of the prognostic five-gene signature in two validation sets

**Figure 3:**
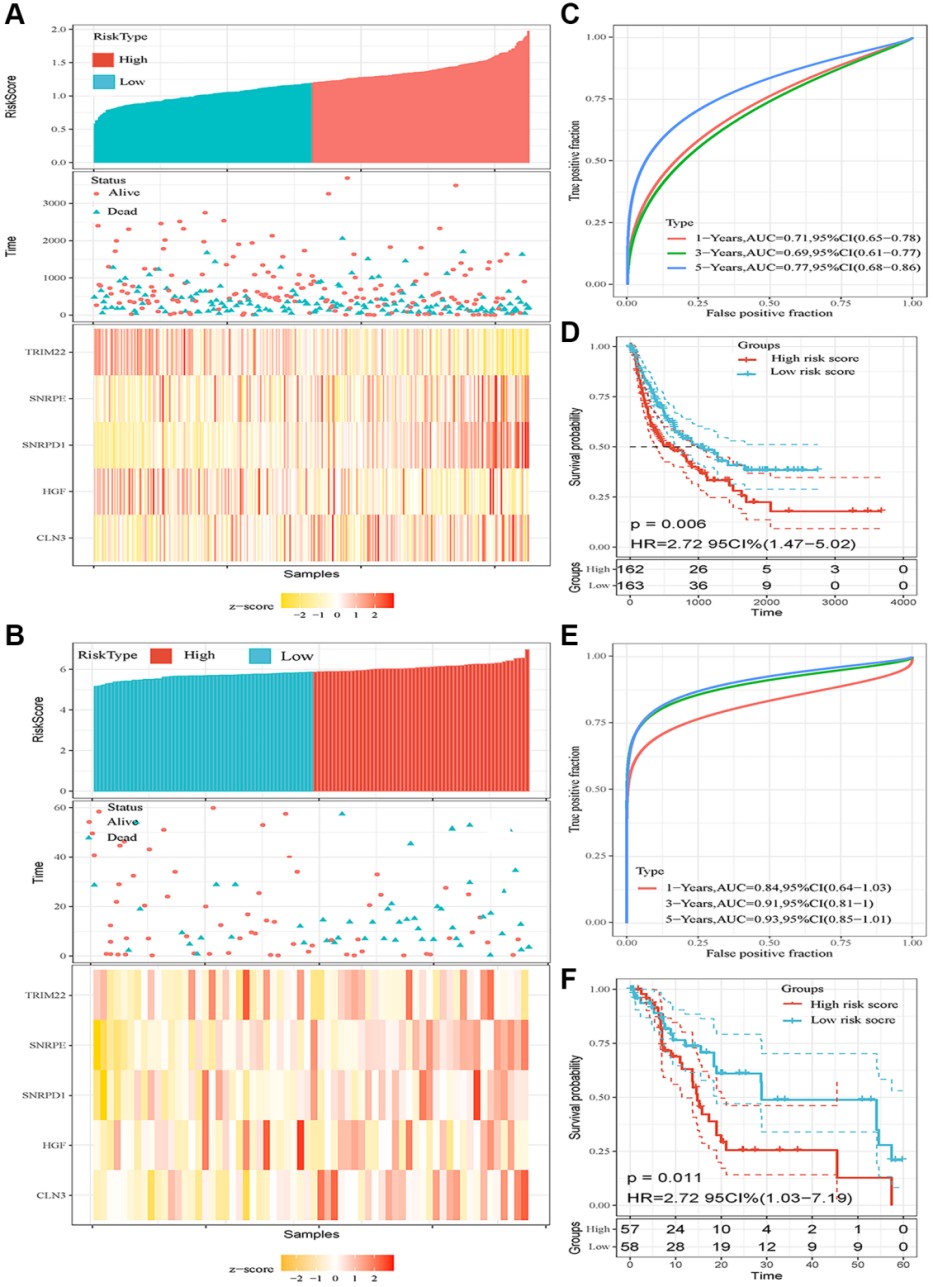
**Prognostic analysis of the five-autophagy-related gene signature model in two validation sets (TCGA HCC cohort and GSE76427 dataset).** (**A**, **B**) Risk score and mRNA expressed heatmap of the five-gene signature in the TCGA HCC cohort (**A**) and GSE76427 dataset (**B**). (**C**) Time-dependent ROC curves of the five-gene signature in the TCGA HCC cohort. (**D**) High-risk score correlated with poor RFS probability in the TCGA HCC cohort. (**E**) Time-dependent ROC curves of the five-gene signature in the GSE76427 cohort. (**F**) High-risk score correlated with poor RFS probability in the GSE76427 cohort.

**Figure 4:**
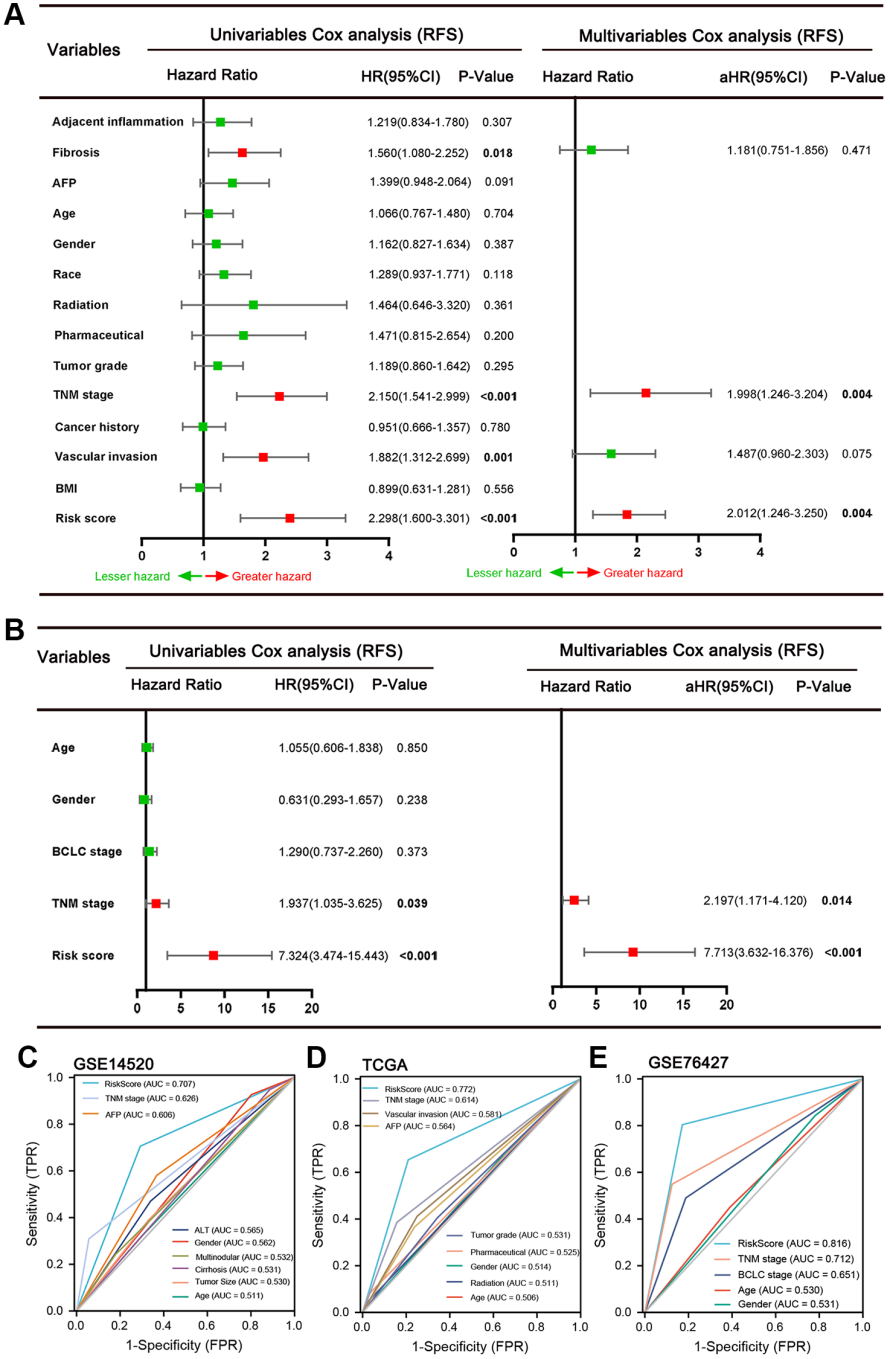
Results of the univariate and multivariate Cox regression analyses regarding RFS in the TCGA (**A**) and GSE76427 (**B**) HCC cohort. ROC curve of GSE14520 (**C**), TCGA (**D**) and GSE76427 datasets (**E**). It was found that the risk score exhibited the largest AUC for RFS in three datasets.

We validated the prediction performance of five ARGs signatures in two independent validation sets (TCGA LIHC cohort and GSE76427 dataset). The HCC patients of two cohorts were classified into two groups based on the risk score, respectively (Figure 3A, 3B). In the TCGA HCC cohort, the AUCs of the time-dependent ROC curve for 1-, 3-, and 5-year RFS were 0.71, 0.69, and 0.77, respectively (Figure 3C). Afterward, the Kaplan–Meier survival analysis exhibited a significantly worse RFS in high-risk patients (*P* = 0.006, Figure 3D). The correlation analysis revealed that high-risk score was associated with increased tumor grade (*P* = 0.002), TNM stage (*P* = 0.002), Serum AFP level (*P* = 0.021), higher recurrence rate (*P* < 0.001), and lower alive rate (*P* < 0.001, Table 3). In the GSE76427 dataset, the AUCs of 1-, 3-, and 5-year RFS were 0.81, 0.91, and 0.93, respectively (Figure 3E). Consistent with the TCGA HCC cohort, the patients in high-risk score groups had shorter RFS than patients in low-risk score groups (*P* = 0.011, Figure 3F). The high-risk score correlated to poor clinicopathological parameters was also observed in the GSE76427 dataset (Table 4). Besides, the high-risk score was also an independent risk predictor of RFS both in the TCGA HCC (Figure 4A) cohort and GSE76427 dataset (Figure 4B). We next compared the predictive capacity of risk scores and other clinical parameters for postoperative recurrence in the follow-up period in three datasets. Results showed that the risk score exhibited the largest AUC for RFS in GSE14520 (Figure 4C), TCGA (Figure 4D) and GSE76427 datasets (Figure 4E).

**Table 3:** Correlation between risk score and clinicopathological features of HCC patients for RFS in the TCGA HCC cohort.

**Characteristics**		***N***	**Risk score level**		***X* ^2^**	^*^P-Value
			**Low**	**High**		
Age	≥60	119	60	59	0.025	0.875
	<60	206	102	104		
Gender	Male	216	105	111	0.393	0.531
	Female	109	57	52		
Race	White	157	87	70	3.766	0.052
	Other	168	75	93		
Tumor grade	G1–G2	204	115	89	9.336	0.002
	G3–G4	121	47	74		
Radiation	Yes	9	4	5	0.108	0.742
	No	316	158	158		
Pharmaceutical	Yes	17	7	10	0.539	0.453
	No	308	155	153		
TNM stage	I–II	238	131	107	9.601	0.002
	III–IV	87	31	56		
Adjacent inflammation	NO	111	58	53	0.130	0.719
	Yes	119	65	54		
Serum AFP level	≥300 ng/ml	48	15	33	5.326	0.021
	<300 ng/ml	224	111	113		
Fibrosis	Yes	106	59	47	1.329	0.249
	No	166	82	84		
Cancer history	No	186	88	98	1.329	0.242
	Yes	97	53	44		
Vascular invasion	NO	181	104	77	2.823	0.093
	Yes	94	44	50		
Recurrence	Yes	153	59	94	14.725	<0.001
	No	172	103	69		
Survival status	Dead	113	40	73	14.464	<0.001
	Alive	212	122	90		

**Table 4:** Correlation between risk score and clinicopathological features of HCC patients for RFS in the GSE76427 dataset.

**Characteristics**		***N***	**Risk score level**		***X* ^2^**	^*^P-Value
			**Low**	**High**		
Age	≥60	67	35	32	0.807	0.369
	<60	49	21	27		
Gender	Male	93	47	46	0.660	0.416
	Female	22	9	13		
BCLC stage	0–A	78	38	40	0.001	0.994
	B–C	37	18	19		
TNM stage	I–II	82	45	37	4.372	0.037
	III–IV	33	11	22		
Recurrence	Yes	51	16	35	11.008	0.001
	No	64	40	24		
Survival status	Dead	23	7	16	3.837	0.050
	Alive	92	49	43		

### Building and validating a nomogram for RFS clinical prediction

**Figure 5:**
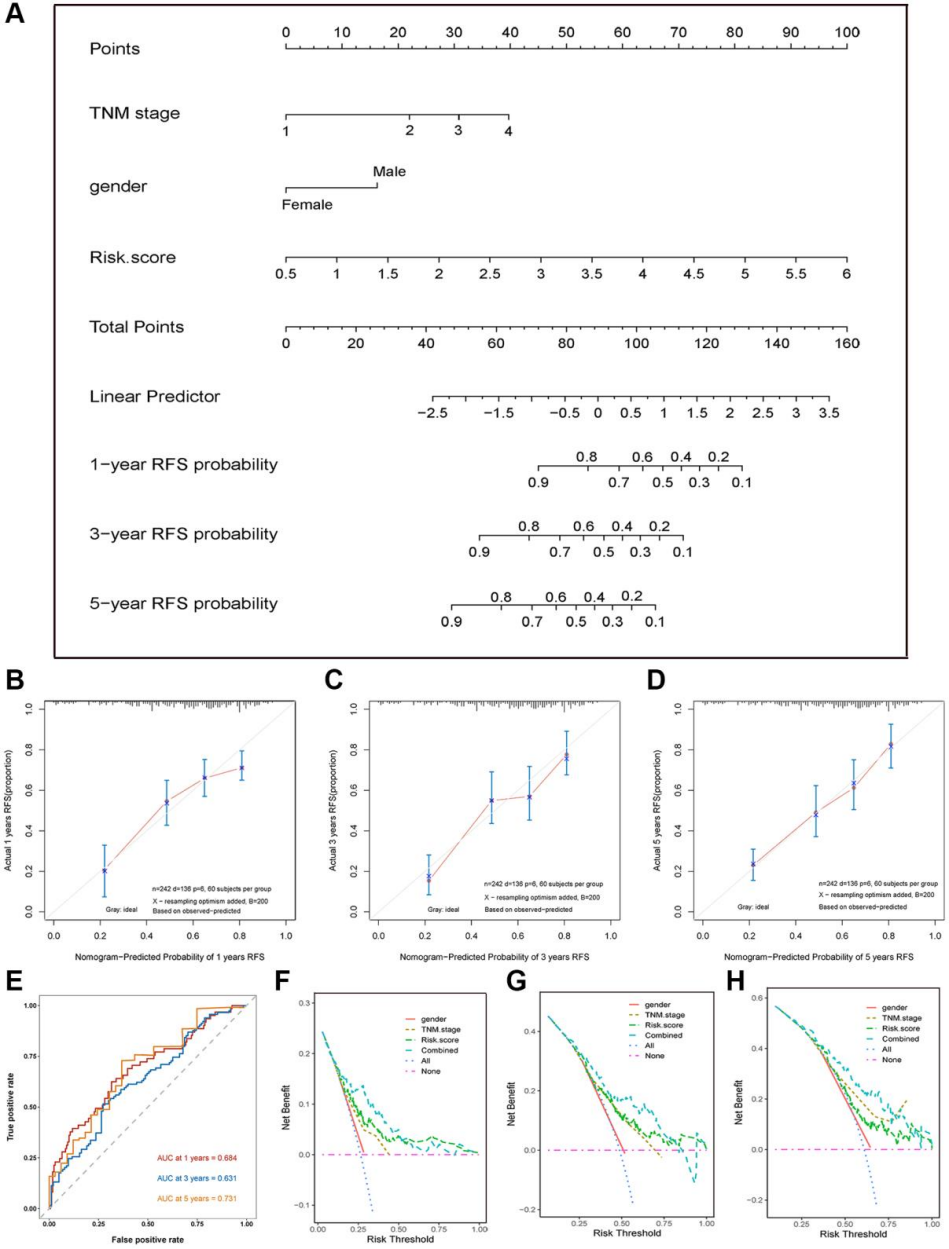
**Nomogram, calibration plot, and DCA curves.** (**A**) Nomogram assembled from the training set to predict 1-, 3-, and 5-year recurrence-free survival probability. (**B**–**D**) The calibration plot of the nomogram for predicting the recurrence-free survival probability at 1- (**B**), 3- (**C**), and 5-year (**D**). (**E**) The time-dependent ROC curves of the nomogram for predicting the recurrence-free survival at 1-, 3-, and 5-year. (**F**–**H**) DCA curve shows that the combined model exhibited the highest net benefit for 1- (**F**), 3- (**G**), and 5-year (**H**) RFS prediction.

We next integrated all the independent predictive factors including gender, TNM stage, and risk score selected by multivariate Cox regression analysis to establish a nomogram (Figure 5A). The C-index of the combined nomogram model was 0.797. The calibration curve at 1-, 3-, and 5-year RFS prediction exhibited the excellent prediction effects of the nomogram (Figure 5B–5D). Furthermore, the time-dependent ROC curve showed that the AUCs of the nomogram model in 1-, 3-, and 5-years RFS prediction were 0.68, 0.63, and 0.73, respectively (Figure 5E). In addition, the DCA curve showed that the combined model exhibited the highest net benefit for 1-, 3-, and 5-year RFS prediction compared with the three single predictive factors (Figure 5F–5H). In summary, all results exhibited the excellent prediction performance of the combined nomogram model for 1-, 3-, and 5-year RFS of HCC patients.

### Genetic alteration was associated with poor RFS probability in HCC patients

**Figure 6:**
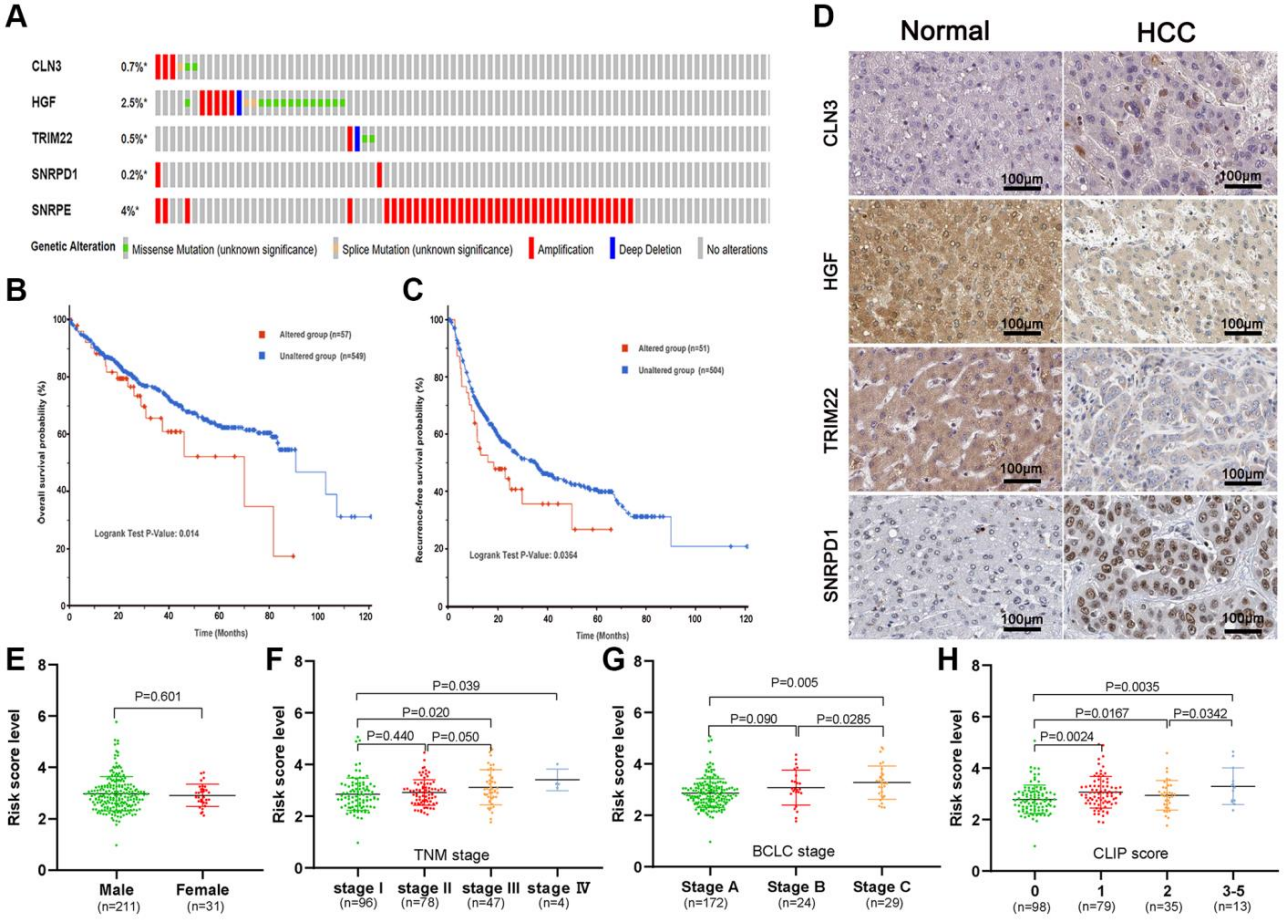
**Genetic alteration and protein expression analysis of five-gene signature in HCC.** (**A**) The summary of genetic alterations of the five-gene signature in the cBioPortal database. (**B**, **C**) The genetic alteration of five-gene signature correlated with poor overall survival probability (**B**) and recurrence-free probability (**C**). (**D**) The representative protein expression of the CLN3, HGF, TRIM22, and SNRPD1 in HCC and non-HCC tissues. (**E**–**H**) Association of the risk score with gender (**E**), TNM stage (**F**), BCLC stage (**G**), and CLIP score (**H**).

We investigated the genetic alteration of the five-ARGs in the cBioPortal database and found that 64 (9.4%) among 673 HCC patients had genetic alterations, most of which were gene amplification (Figure 6A). In addition, patients with genetic alteration had shorter OS (*P* = 0.014) and RFS (*P* = 0.036) than patients without genetic alterations (Figure 6B, 6C). We next investigated the dissimilarity of protein expression of five genes between the HCC tissues and the non-HCC tissues in the Human Protein Atlas database. We found that CLN3 and SNRPD1 were highly expressed, while HGF and TRIM22 were lowly expressed in HCC tissues (Figure 6D). However, SNRPE was not found on the website. Then, we compared the risk score between different stage HCC patients in the GSE14520 dataset to investigate the differentially predictive performance of the gene signature. The risk score was incrementally increased with increasing TNM stage, BCLC stage, and CLIP score, but doesn’t associate with genders (Figure 6E–6H).

### Functional enrichment analysis via GO, KEGG, and GSEA

**Figure 7:**
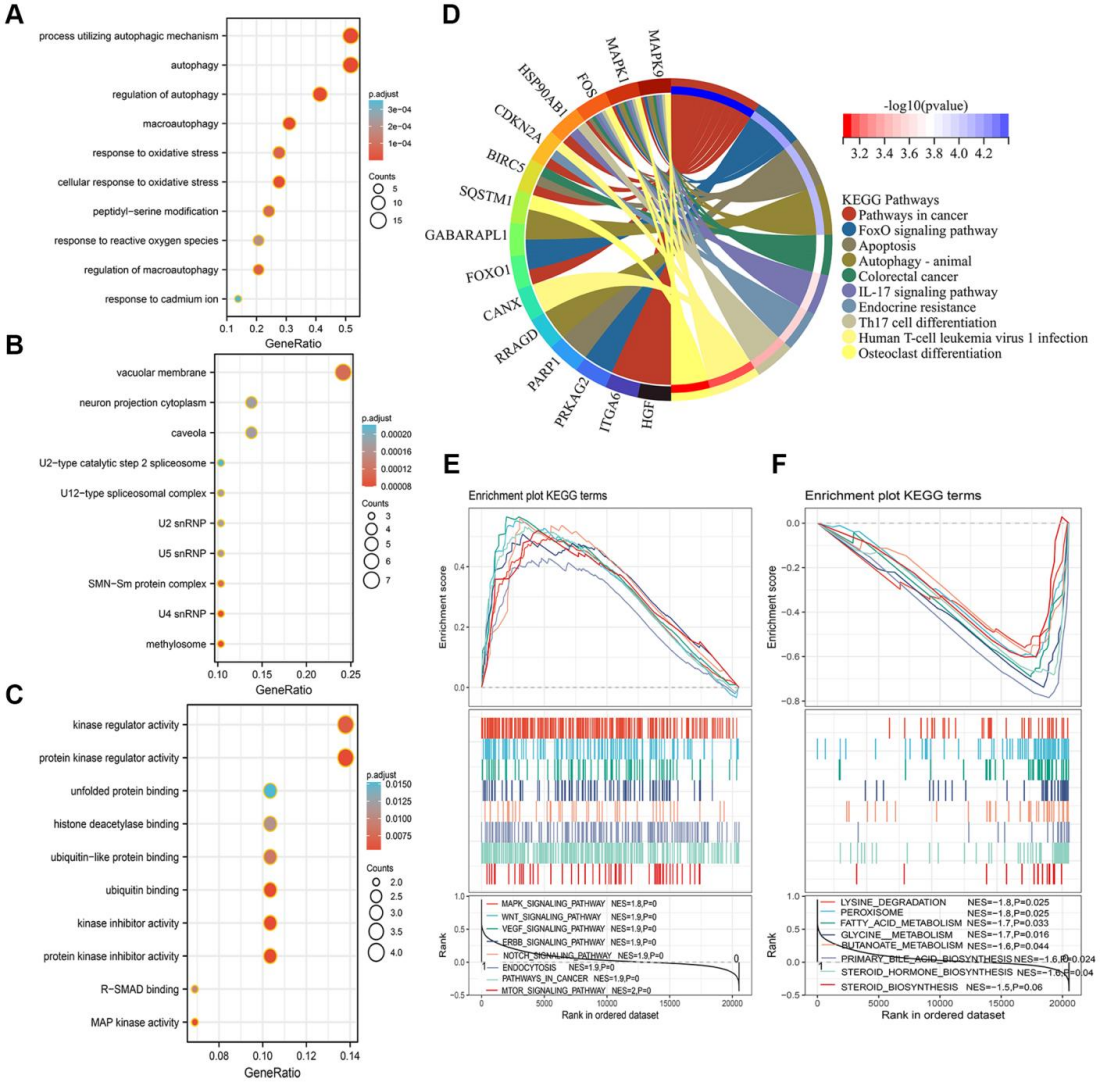
**Functional analysis of GO, KEGG, and GSEA.** (**A**–**D**) The 29 autophagy-related differentially expressed genes were mainly enriched in the biological process (**A**), cellular component (**B**), molecular function (**C**), and KEGG pathway (**D**). (**E**, **F**) Identification of significant signaling pathway enriched in the high-risk group (**E**) and low-risk group (**F**) by GSEA.

The 29 ARGs were used to perform the GO and KEGG enrichment analysis to elucidate the biological functions and pathways. These ARGs were significantly enriched in the autophagy-related biological process, such as autophagy, response to oxidative stress, and regulation of autophagy (Figure 7A). In the cellular component, the ARGs were related to vacuolar membrane, neuron projection cytoplasm, and caveola, etc., (Figure 7B). In the molecular function, the ARGs were mainly related to kinase regulator activity, unfolded protein binding, and histone deacetylase binding, etc., (Figure 7C). The KEGG analysis indicated that ARGs were mainly enriched in pathways in cancer, FoxO signaling pathway, autophagy, and other cancer-related pathways (Figure 7D). Next, GSEA analysis was implemented to explore the significant signaling pathway that high- and low-risk score patients enriched. Results showed that high-risk score patients were associated with cancer regulating related pathways, such as mTOR signaling pathway, WNT signaling pathway, and VEGF signaling pathway, etc., (Figure 7E). Meanwhile, the low-risk score patients were negatively related to Lysing degradation, Peroxisome, Fatty acid metabolism, and Primary bile acid biosynthesis, etc., (Figure 7F).

### Differential abundance analysis of tumor-infiltrating immune cells in two risk score groups

**Figure 8:**
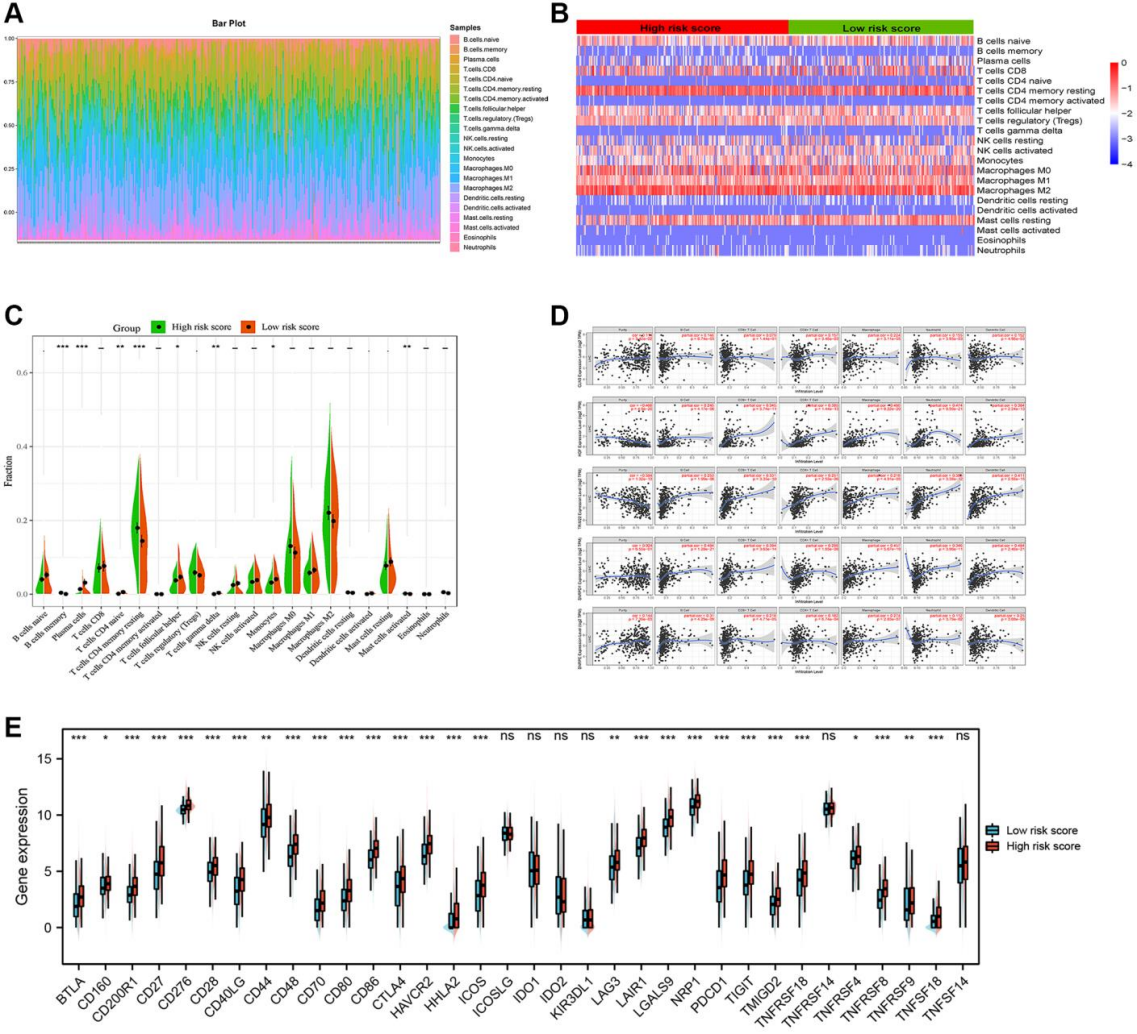
**Immune infiltration analysis.** (**A**) Estimation of fractions of immune cells of each tissue, where different colors represented different immune cells. (**B**) The heatmap showed the infiltration difference of immune cells between the high-risk and low-risk score groups. (**C**) The comparison of estimated fractions of 22 immune cells between the high-risk and low-risk score groups. (**D**) Five ARGs were associated with tumor purity or seven important immune cells. (**E**) Most of immune checkpoint related genes were highly expressed in the high-risk score group.

We calculated the estimated fractions of 22 immune cells in each HCC tissue using the CIBERSORT algorithm and we excluded the samples with the *P* value > 0.05 to guarantee the accuracy of the analysis. The sum of the estimated fraction of 22 immune cells in each HCC tissue was one. Then, we visualized the fractions of each tissue in a box plot, and different colors represented different immune cells (Figure 8A). Next, we analyzed the associations between gene signature and estimated fractions of 22 immune cells. The heatmap showed the infiltration difference of immune cells between the high-risk and low-risk score groups (Figure 8B). In addition, the Wilcoxon rank-sum test demonstrated that high-risk score groups have a higher level of immune cells in the tumor microenvironment, such as B cells memory, T cells CD4 memory resting and mast cells activated (Figure 8C). Furthermore, the correlations analysis revealed that five ARGs were associated with tumor purity or seven important immune cells, especially HGF, TRIM22, and SNRPD1 (Figure 8D). Next, we investigated the expression of immune checkpoints related genes and found that most of these genes, such as BTLA, PDCD1 and CTLA4 were highly expressed in the high-risk score group (Figure 8E). So, it was a reasonable hypothesis that the high-risk score group correlated with a higher degree of immune infiltration, whereas the high expression of immune checkpoint genes causes a low response in the immune state, suggesting that HCC patients in the high-risk score group may get more benefit from immune checkpoint blockers.

### SNRPE regulates the proliferation and migration of HCC cells

**Figure 9:**
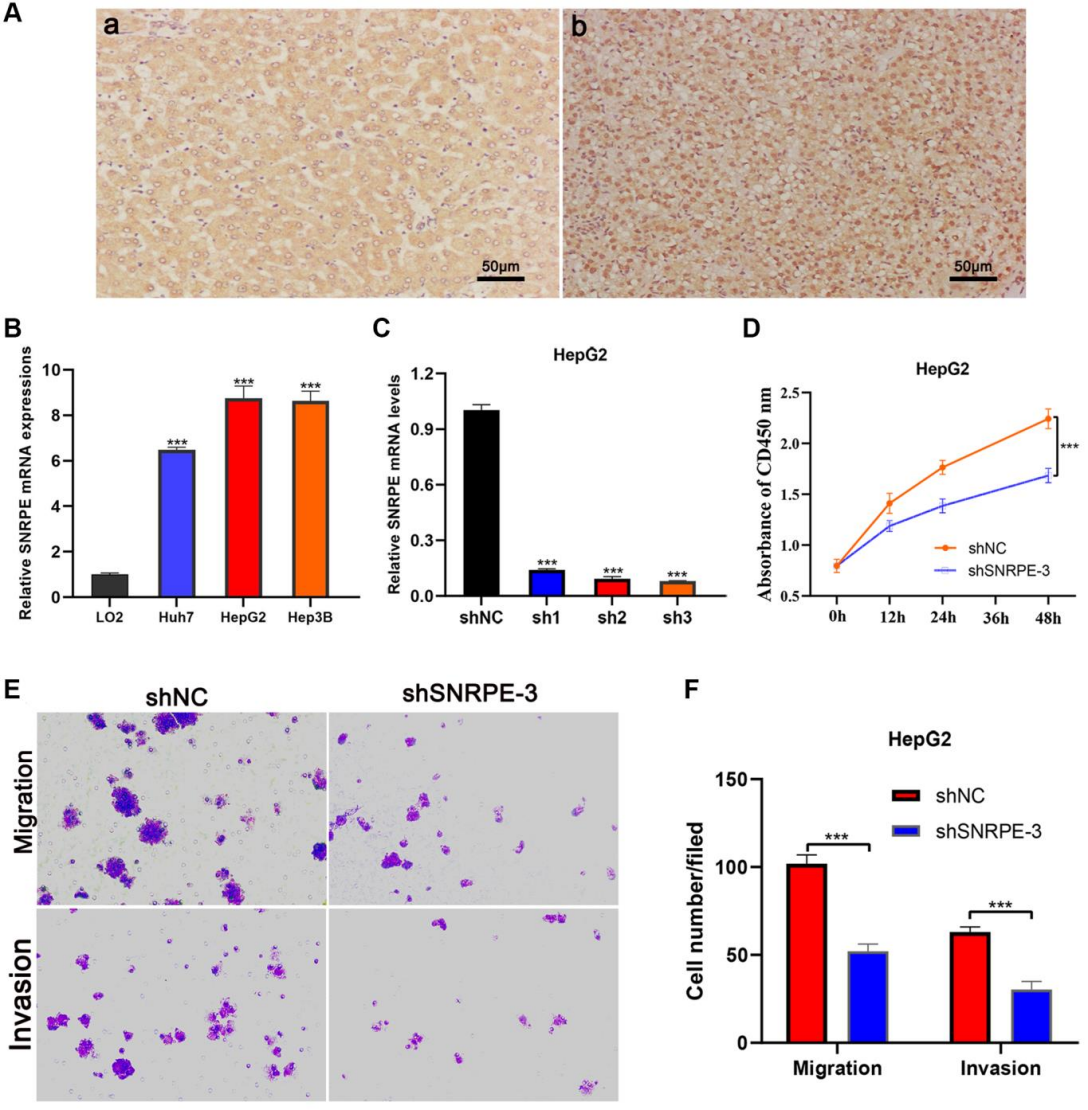
**Immunohistochemistry and cell experiments of gene SNRPE to validate our results.** (**A**) Compared with adjacent normal liver tissues (**a**), SNRPE protein expression was significantly overexpressed in HCC tissues (**b**) (×200 magnification). (**B**) SNRPE mRNA expression was greater in HCC cells than in the normal liver cells. (**C**) The shSNRPE with lentiviral transfection was transfected into HepG2 cells. (**D**) CCK8 assay. After SNRPE knockdown, the HepG2 cells exhibited a significant inhibition in viability. (**E**, **F**) Transwell assay. After SNRPE knockdown, the HepG2 cells exhibited a significant inhibition in migration and invasion.

In multivariate Cox regression analysis, the aHR value of SNRPE was the highest, its role in HCC has not yet been elucidated, so we conducted cellular and molecular biology assays to investigate the role of SNRPE in the progression of HCC. As shown in the representative figures, SNRPE protein expression was significantly higher in HCC tissues than in adjacent normal liver tissues (Figure 9A). We next investigated the SNRPE mRNA expression in normal hepatocyte cell line LO2 and three HCC cell lines (Huh7, HepG2, Hep3B) and found that SNRPE was significantly overexpressed in three HCC cell lines (Figure 9B). Then, the shSNRPE with lentiviral transfection was transfected into HepG2 cells and qRT-PCR affirmed that SNRPE mRNA expression was remarkably inhibited (Figure 9C). The shSNRPE-3 was selected for subsequent experiments due to its maximum inhibitory effect. CCK8 assay revealed that after SNRPE knockdown, the HepG2 cells showed a remarkable reduction in proliferation (Figure 9D). In addition, transwell assay further showed that the migration and invasion capacity of HepG2 cells also significantly decreased after SNRPE knockdown (Figure 9E, 9F).

## DISCUSSION

Although the increase in the number of HCC patients is slowing in recent years, the morbidity and mortality are still high in high HBV or HCV incidence countries, especially sub-Saharan and East Asia [20, 21]. Studies reported that more than 70% of patients undergoing radical resection will recur within 5 years [7, 22]. Therefore, building an effective model to predict postoperative recurrence and identify HCC patients with short RFS was essential for clinical decision and prognostic improvement. The increasing number of research validated that autophagy plays a prominent role in the tumorigenesis and progression of HCC [23–25]. Abnormal expression of ARGs has also been confirmed to be related to the overall survival of HCC [26, 27], but there has been rare research focus on the role of multiple ARGs in RFS.

In this present study, we built a novel autophagy-related five-gene signature according to the relative transcription level (including CLN3, HGF, TRIM22, SNRPD1, and SNRPE) in the GSE14520 dataset (training set) for RFS prediction in patients with HCC. The Kaplan–Meier survival analysis and time-dependent ROC curve both exhibited the excellent prediction performance of the five-ARG signature. In addition, the correlation analysis illuminated that the high-risk score based on five-ARG signature transcription levels was associated with worse clinicopathological parameters, such as TNM stage, serum AFP level, CLIP score, BCLC stage, and survival status. Moreover, the multivariate Cox regression analyses affirmed that high-risk score was an independent predictor of RFS. We then validated the reliability and prediction performance of gene signature in two independent HCC cohorts (TCGA HCC cohort and GSE76427 dataset). Compared with existing signatures, our prognostic model exhibited better performance to predict the RFS of HCC patients [28, 29].

To accurately and easily predict the 1-, 3-, and 5-year RFS of HCC patients, a nomogram integrating the multigene signature and all independent predictors identified by multivariate Cox regression analyses was constructed. The C-index of the combined nomogram model was 0.797. The calibration curve suggested that there was an excellent consistence between the nomogram prediction and actual observation. In addition, the DCA curve shows the highest net benefit of the combined model for 1-, 3-, and 5-year RFS prediction. In this study, we also investigated the genetic alteration of the five-ARG signature and found that genes alteration related to worse OS and RFS. So, we speculated that the genetic alteration may be a driver for the abnormal expression and tumor progression. Moreover, the function analysis by GO and KEGG revealed that the ARGs were significantly enriched in the autophagy-related pathways and other HCC progression-related pathways, such as Hepatitis B, FoxO signaling pathway, and Pathways in cancer [30]. Furthermore, the GSEA analysis suggested that high-risk score patients were associated with the mTOR signaling pathway, which plays a crucial role in regulating autophagy [31, 32]. GSEA identifies functions that are not related to autophagy but to cancer-promoted pathways due to a small number of autophagy-related genes, which might explain the underlying molecular mechanisms of these ARGs.

Besides, we further investigated the relationship between the risk score and the immune infiltrate in the TCGA HCC cohort and found that the high-risk group has a higher level of immune cells in the tumor microenvironment, such as B cells memory, T cells CD4 memory resting and mast cells activated. In addition, most of immune checkpoints related genes were highly expressed in the high-risk score group. So, it was a reasonable hypothesis that the high-risk score group correlated with a higher degree of immune infiltration, whereas the high expression of immune checkpoint genes causes a low response in the immune state, suggesting that HCC patients in the high-risk score group may get more benefit from immune checkpoint blockers.

According to our results, five autophagy-related genes (CLN3, HGF, TRIM22, SNRPD1, and SNRPE) were independent risk factors of RFS. CLN3 has been found frequently upregulated in HCC tissues and cell lines and was significantly correlated with worse clinicopathological parameters and poor prognosis [33]. In addition, Zhong et al. reported that deletions of CLN3 increased autophagic flux, suppressed mTORC1 and Akt activities, suggesting autophagy induction [34]. Hepatocyte growth factor (HGF), produced by stromal cells, plays an essential role in stimulating epithelial cell proliferation, motility, morphogenesis, and angiogenesis in various organs [35]. Bell and colleagues uncovered that autophagy regulates degradation of Met/HGF and HGF-dependent cell migration and invasion [36]. In addition, previous studies demonstrated that HGF/Met signaling pathway was strongly linked with the tumorigenesis and progression of HCC [37, 38]. Moreover, HGF in peritumoral liver tissue has been reported as a major risk factor of HCC recurrence, which was consistent with our result [39]. TRIM22, a member of tripartite motif family protein which is involved in autophagy and innate immunity process, was downregulated in various types of tumors and associated with a poor prognosis [40, 41]. For the first time, we reported that TRIM22 was an independent predictor for RFS of patients with HCC. SNRPD1 and SNRPE, the crucial genes encoding core spliceosome components, were abnormally highly expressed in HCC tissues [42, 43]. Quidville et al. indicated that SNRPE or SNRPD1 targeting mediates the death of SKBr-3 cell lines through autophagy [18]. Cellular and molecular biology assays demonstrated that SNRPE overexpressed in HCC cells and regulated the proliferation and migration of HepG2 cells. The multivariate Cox regression analysis demonstrated that the aHR value of SNRPE was the highest among of 5 ARGs. So, SNRPE plays the main role in the 5-gene signature for the clinical RFS prediction. The clinical prognostic value of individual SNRPE genes have not been investigated and we speculated that SNRPE has the clinical prognostic role for HCC, which was the main direction of our research in the near future.

To our knowledge, the five-ARG signature and nomogram for RFS prediction have not been reported previously and could be a practical and accurate prognostic classification tool of HCC. However, basic experimental was required to address the detailed mechanism of each ARG involved in the tumorigenesis and progression of HCC. Nonetheless, our study also has some limitations. First, although the 5-gene signature and the predictive nomogram were built and validated by different databases and validated by the IHC and cell experimental, the specific functions and molecular mechanisms should be investigated by various *in vitro* and *in vitro* approaches. Second, some factors that cannot be ignored in HCC development such as hepatitis B and C were not included in our research, and need further clinical trials.

Collectively, this study defined a novel gene signature and generated a combined nomogram model to predict RFS after hepatectomy, and provided a tool to help the clinical decision-making for personalized treatment in HCC patients. There were correlations existed for autophagy-related genes and tumor immune infiltration, but warrant further investigation.

## METHODS

### Acquisition of mRNA expression data and clinical characteristics

Transcription profiling mRNA data and corresponding clinical data were downloaded from the GEO database (GSE14520 and GSE76427 dataset) and the TCGA database. The raw data downloaded in TCGA were normalized RNA-sequencing data as transcripts per million (TPM). For GSE14520 and GSE76427 datasets, the microarray data sets were transformed into TPM values and underwent a log2 transformation and the batch effect in different datasets was removed. The autophagy-related genes (ARGs) were obtained from the Human Autophagy Database (HADb) and searched in PubMed. The multi-genes signature was constructed using the data of the GSE14520 dataset (training set) and validated using the data of the TCGA HCC cohort and GSE76427 dataset (validation set). The samples in TCGA, GSE14520 and GSE76427 datasets that met the following inclusion criteria were included in this study: (1) All samples had mRNA sequencing data; (2) Included patients had been pathologically confirmed with HCC; (3) The clinicopathological parameters of included patients were relatively complete, including TNM stage, recurrence-free survival time, etc.; (4) Follow-up period > 30 days. It was not needed for additional ethical approval in this research, because of the acquisition of all transcription profiling mRNA data and corresponding clinical data from the publicly available database.

### Differentially expressed analysis of ARGs

We used the limma R package to perform the differentially expressed gene analysis using transcription profiling mRNA data in the training set (GSE14520). Differentially expressed genes with absolute log2 fold change (FC) > 1 and an adjusted *P*-value < 0.05 were considered for next step analysis. Then, the overlapping gene between the ARGs obtained from the HADb and differentially expressed genes identified from the training set were screened out as differential expressed ARGs and used for subsequent analysis.

### Construction and validation of the autophagy-related gene signature

We conducted the Univariate and Multivariate Cox proportional hazards regression mode on differential expressed ARGs to identify the gene that independently predict the RFS of HCC patients. Next, these genes were used to construct the prognostic gene signature. We then calculated the risk score of each patient of the GSE14520 dataset. The risk score was calculated based on the following formula: Risk score = (β_mRNA1_ × expression level of mRNA1) + (β_mRNA2_ × expression level of mRNA2) + (β_mRNA3_ × expression level of mRNA3) + … + (β_mRNAn_ × expression level of mRNAn). The patients were stratified into low-risk score group and high-risk score group based on the cutoff value of the median score. In addition, we performed the time-dependent receiver operating characteristic (ROC) curves and Kaplan–Meier survival analysis to assess the prediction capacity of a prognostic gene signature for RFS. Furthermore, the gene signature and some clinical characteristics such as age, gender, TNM stage, tumor grade and serum AFP level were integrated to perform the Cox regression analysis for identification of the independent predictors of RFS. The characteristics with *P* < 0.05 from the univariate analysis were subsequently used for the further analysis in the multivariate Cox’s proportional hazard model with forward LR model. Finally, we validated the prediction performance of gene signature in the independent HCC cohort of TCGA and GSE76427 dataset. We calculated the risk scores with the same formula for each patient in two datasets.

### Establishing and validating a predictive nomogram

All independent risk factors of RFS in the training set, including the risk score model and clinical characteristics identified by the multivariate Cox regression analysis, were integrated to establish a nomogram that has the better prediction performance of 1-, 3-, and 5-year RFS. We used the “survival” R package to calculate Harrell’s concordance index (C-index) to evaluate the discrimination of the nomogram. Next, calibration curves of RFS probability at 1-, 3-, and 5-year were plotted to discriminate the probabilities predicted by the nomogram and actually. Then, the “timeROC” R package was utilized to plot the time ROC to evaluate the prediction accuracy of the nomogram. The “ggDCA” R package was used to plot the decision curve analysis (DCA) curve to help make the clinical decision for the acquisition of the best net benefit.

### Genetic alteration, protein differential expression analysis

The accumulation of genetic alterations can drive cancer progression by inducing abnormal genes expression [44]. We selected two liver Hepatocellular Carcinoma datasets (TCGA-Firehose Legacy and AMC-Hepatology 2014) for a total of 673 HCC patients in the cBioPortal database to investigate the affection of genetic alteration of gene signature on survival probability [45]. We next investigated the dissimilarity of protein expression of gene signature between the HCC tissues and the non-HCC tissues in the Human Protein Atlas database to further analyze the affection of abnormal protein expression of gene signature on survival probability.

### Functional enrichment analysis

The differential expressed ARGs were uploaded to the DAVID database to perform the GO and KEGG enrichment analysis to explore the potential mechanism that these ARGs regulate the tumorigenesis and progression of HCC [46]. mRNA expression data in the GSE14520 dataset were uploaded to the GSEA software (version 4.1.0) to perform GSEA enrichment analysis. We stratified the 242 HCC patients into low-risk score group and high-risk score group taking the cutoff value of the median risk score. The enriched pathway items with adjusted *p*-value < 0.05 and false discovery rate (FDR) *q*-value < 0.25 were selected.

### Immune infiltration analysis of gene signature

CIBERSORT, using gene expression data to provide an estimation of the abundances of member cell types in a mixed cell population, was used to investigate the relationship between the gene signature and the immune infiltrate [47]. After calculation and filtration with *P* < 0.05, the bar plot was plotted to exhibit the proportions of different immune cells in each HCC sample. Then, we plotted the heatmap of 22 types of infiltrating immune cells of each HCC sample. In addition, we compared the differential abundance of immune infiltrate between high-risk score groups and low-risk score groups. Furthermore, we investigated the correlations between gene signature with several important immune cells (B cells, CD4+ T cells, CD8+ T cells, Neutrophils, Macrophages, and Dendritic cells) in the TIMER web server [48]. We then investigated the expression of immune checkpoint genes between the high and low-risk groups and visualized in a boxplot. The list of immune checkpoint genes was determined from previous relevant articles [49, 50].

### Immunohistochemistry (IHC) assay

Three fresh HCC specimens and paired adjacent tissues were obtained from the Department of Hepatobiliary Surgery, 900 Hospital of the Joint Logistic Team. The patients must meet the following criterions: only one tumor node and no metastasis, Child-Pugh class A, no cancer radiotherapy or chemotherapy history before the operation, postoperative pathology confirmed as HCC. These samples were made into formalin-fixed paraffin-embedded blocks and then were cut into 4-μm sections. Immunohistochemistry staining was accomplished as described earlier [51]. The specific antibodies were used as follows: SNRPE (PA5-96342; 1:300; Thermo Fisher Scientific, USA); secondary antibody (1:50,000; KIT-5010; anti-rabbit/mouse IgG; China Fuzhou Maixin Biotechnology Development Co., Ltd.). The sections were stained with 3,3′-diaminobenzidine and substrate chromogen (Dako) and then counterstained with hematoxylin. The study was approved by the Human Subjects Protection Committee of the 900 Hospital of the Joint Logistic Team. Furthermore, we obtained written informed consent from all participants prior to surgery. All experiments were performed in accordance with relevant guidelines and regulations.

### Cell culture and transfection

The normal hepatocyte cell line LO2 and HCC cell lines Huh7, HepG2, Hep3B were obtained from the Chinese Type Culture Collection of the Chinese Academy of Sciences (Shanghai, China). We cultured these cell lines in DMEM (Gibco) supplemented with 10% heat-inactivated fetal bovine serum (FBS, Gibco, 10099141). All these cells were maintained in an incubator under a moist atmosphere of 5% CO2 at 37°C. The HepG2 cells were transfected with shSNRPE or sh-NC using Lipofectamine™ 3000 Transfection Kit (L3000015, Invitrogen, USA), according to the manufacturer’s protocol. The shRNA sequences for SNRPE were as follows: sense strand sequence for shSNRPE: 5′-GCTCTATGAGCAAGTGAAT-3′.

### Quantitative polymerase chain reaction (qRT-PCR)

According to the manufacturer’s instruction, we extracted the total RNA using RNAiso Plus (TaKaRa, 9109, China) and then reverse-transcribed it into cDNA for subsequent PCR assay using gDNA Purge (Novoprotein, E047-01A, China). The reverse transcription and qRT-PCR methods were accomplished as described earlier. Glyceraldehyde-3-phosphate dehydrogenase (GAPDH) was used as the internal control for SNRPE. The 2−ΔΔCt method was used to determine the relative quantification of SNRPE. The sequence of primer pairs was designed as following: SNRPE (forward: 5′-ACCATGGCGTACCGTGGC-3′, reverse: 5′-CTAGTTGGAGACACTTTGTAGCAGA-3′); GAPDH (forward: 5′-TTGGCTTGACTCAGGATTTA-3′, reverse: 5′-ATGCTATCACCTCCCCTGTG-3′).

### CCK-8 assay

The HepG2 cell viability was detected by the cell counting Kit-8 (CCK-8) method. After siRNA transfected 48 h, HCC cells were added to each well of the plate. Then, the CCK-8 (MA0218, Meilune, Dalian, China) reagent was added to each well and the plate was subsequently incubated in the incubator in dark conditions. Finally, the absorbance at a 450-nm wavelength was employed to determine the viable cells.

### Transwell migration and invasion assays

We performed the transwell assays using 24-well transwell plates (Corning Inc., Corning, NY, USA) to assess the migration and invasion capacity of HepG2 cells. For migration assays, transfected cells were resuspended in a serum-free medium and seeded in the upper chamber, and 700 μl of 15% FBS medium was also added to the bottom of the chamber. For invasion assays, DMEM-diluted Matrigel (BD Biosciences, USA) was precoated on the 24-well transwell plates before transfected cells were seeded. Subsequently, the migrating and invading cells under the surface of the membrane were fixed with 95 % methanol and stained with crystal violet (MA0150, Meilune, Dalian, China) for 30 min.

### Statistical analysis

The R software (version 4.1.0) and related packages were employed to perform the statistical analysis and plotted. Chi-squared test was used to compare the clinicopathological characters between the high and low-risk score group. The predictive factors of RFS were identified by the Univariate and Multivariate Cox regression analyses. K-M method and the log-rank test were used to compare the survival probability between the two groups. The prediction performance of the gene signature and nomogram was assessed by the area under the curve (AUC) of time-dependent ROC. Student’s *t*-test and the Wilcoxon rank-sum test were utilized for subgroups differential analyses. *P* < 0.05 was considered to be statistically significant.

### Data availability

The datasets generated for this study can be found in the GEO database (https://www.ncbi.nlm.nih.gov/geo/) and TCGA database (https://portal.gdc.cancer.gov).
